# Postmenopausal Breast Cancer Is Associated with Exposure to Traffic-Related Air Pollution in Montreal, Canada: A Case–Control Study

**DOI:** 10.1289/ehp.1002221

**Published:** 2010-10-06

**Authors:** Dan L. Crouse, Mark S. Goldberg, Nancy A. Ross, Hong Chen, France Labrèche

**Affiliations:** 1 Department of Geography and; 2 Department of Medicine, McGill University, Montreal, Quebec, Canada; 3 Division of Clinical Epidemiology, McGill University Health Centre, Montreal, Quebec, Canada; 4 Department of Epidemiology, Biostatistics, and Occupational Health, McGill University, Montreal, Quebec, Canada; 5 Département de médecine sociale et préventive et Département de santé environnementale et santé au travail, Université de Montréal, Montreal, Quebec, Canada

**Keywords:** air pollution, case–control study, Montreal, nitrogen dioxide, postmenopausal breast cancer

## Abstract

**Background:**

Only about 30% of cases of breast cancer can be explained by accepted risk factors. Occupational studies have shown associations between the incidence of breast cancer and exposure to contaminants that are found in ambient air.

**Objectives:**

We sought to determine whether the incidence of postmenopausal breast cancer is associated with exposure to urban air pollution.

**Methods:**

We used data from a case–control study conducted in Montreal, Quebec, in 1996–1997. Cases were 383 women with incident invasive breast cancer, and controls were 416 women with other incident, malignant cancers, excluding those potentially associated with selected occupational exposures. Concentrations of nitrogen dioxide (NO_2_) were measured across Montreal in 2005–2006. We developed a land-use regression model to predict concentrations of NO_2_ across Montreal for 2006, and developed two methods to extrapolate the estimates to 1985 and 1996. We linked these estimates to addresses of residences of subjects at time of interview. We used unconditional logistic regression to adjust for accepted and suspected risk factors and occupational exposures.

**Results:**

For each increase of 5 ppb NO_2_ estimated in 1996, the adjusted odds ratio was 1.31 (95% confidence interval, 1.00–1.71). Although the size of effect varied somewhat across periods, we found an increased risk of approximately 25% for every increase of 5 ppb in exposure.

**Conclusions:**

We found evidence of an association between the incidence of postmenopausal breast cancer and exposure to ambient concentrations of NO_2_. Further studies are needed to confirm whether NO_2_ or other components of traffic-related pollution are indeed associated with increased risks.

Breast cancer has the highest incidence rate of all cancers in women and is the second leading cause of death from cancer in both Canada (Canadian Cancer Society 2009) and the United States (American Cancer Society 2009). Accepted risk factors for breast cancer include genetic mutations, family history of breast cancer, aspects of reproductive history, and lifestyle factors, such as alcohol consumption. Only about one-third of new cases of breast cancer are attributable to known risk factors, and much of the etiology remains unexplained (Coyle 2004). There have been consistent findings of higher rates of breast cancer in urban areas compared with rural areas, in both Canada (Bako et al. 1984) and the United States (Hall et al. 2005; Reynolds et al. 2004).

Local vehicular traffic is the primary contributor to air pollution in urban areas. Vehicular emissions include gases, particles, volatile organic compounds, and polycyclic aromatic hydrocarbons (PAHs), many of which are accepted or potential carcinogens. Benzene, for example, is present in gasoline, is an accepted human carcinogen (International Agency for Research on Cancer 1987), and has been shown to cause mammary carcinomas in rodents (Huff et al. 1989; Maltoni et al. 1988, 1989). Exposure to other aromatic hydrocarbons associated with gasoline, including kerosene, toluene, and xylenes, has also produced increased rates of mammary cancers in female rats (Maltoni et al. 1997). Aromatic hydrocarbons are lipophilic and may therefore reach elevated concentrations in breast tissue and promote carcinogenesis in the cells of the breast (Morris and Seifter 1992). We postulated in the mid-1990s (Labrèche and Goldberg 1997) that exposure to organic solvents and other lipophilic toxics may cause breast cancer. Santodonato (1997) concluded that, regarding the etiology of human breast cancer, current scientific literature provides “persuasive evidence for the hypothesis that certain carcinogenic PAHs produce a unique duality of pathologic effects encompassing both genotoxic and nongenotoxic components.”

Several studies have shown associations between the incidence of breast cancer and occupational exposure to benzene and to PAHs (Gammon et al. 2002; Labrèche et al. 2010; Petralia et al. 1999). Given that these same pollutants are present in vehicular exhaust and thus present in urban air pollution, it is plausible that traffic-related exposures may contribute to the incidence of breast cancer. A study conducted in Nassau and Suffolk Counties, New York State (Lewis-Michl et al. 1996), suggested a possible increased risk of breast cancer among postmenopausal women living near areas characterized by relatively high traffic compared with other areas [adjusted odds ratio (OR), 1.29; 95% confidence interval (CI), 0.77–2.15]. In a case–control study in Erie and Niagara Counties, New York State, Bonner et al. (2005) used observations from fixed-site pollution monitors to estimate exposure to total suspended particulates and found that early-life exposures to relatively high concentrations (i.e., > 140 μg/m^3^) were associated with an increased risk of developing postmenopausal breast cancer (OR = 2.42; 95% CI, 0.97–6.09) compared with exposure to relatively low concentrations (i.e., < 84 μg/m^3^). In a second analysis from that study (Nie et al. 2007), estimates of residential exposures to benzo[*a*]pyrene, derived from a traffic emissions model, showed an OR of 2.57 (95% CI, 1.16–5.69) for postmenopausal women exposed to higher concentrations at the time of first giving birth compared with those exposed at lower concentrations. They found no evidence that exposures at other periods were associated with increased risk. Furthermore, in analyses stratified by smoking status, statistically significant associations were limited to lifetime nonsmokers.

The purpose of the present study was to determine whether the incidence of postmenopausal breast cancer in Montreal, Quebec, was associated with exposure to intraurban concentrations of nitrogen dioxide (NO_2_), a marker for traffic-related pollution.

## Materials and Methods

Montreal is the second largest city in Canada, with the greater Montreal area having a population > 3.6 million people. Our study is restricted to the Island of Montreal and Nun’s Island, which have a population of approximately 1.8 million people (Statistics Canada 2006).

### Design of the case–control study

We conducted a hospital-based case–control study of incident, invasive cases of postmenopausal breast cancer (Labrèche et al. 2003, 2010). The target population comprised postmenopausal women, 50–75 years of age at the time of diagnosis, who in 1996 and 1997 were residents of the greater Montreal area. Eligible case subjects were diagnosed with primary, invasive breast cancer [*International Classification of Diseases, 9th Revision*, code 174 (World Health Organization 1975)] that was confirmed histologically. Cases were identified from all 18 hospitals in the region that treated breast cancer, thus ensuring almost complete coverage of the target population. To minimize the potential for recall bias, control subjects had one of 32 other selected sites of incident, histologically confirmed cancers. The controls were matched to the cases by hospital and approximately frequency matched by age. The data were collected originally for a study examining risk of breast cancer associated with occupational exposures to selected substances; thus, selected sites of cancer (i.e., liver and intrahepatic bile duct, pancreas, lung, bronchus and trachea, brain and central nervous system, leukemias, and lymphomas) were excluded because of their possible association with occupational exposures. The controls were approximately frequency matched to the cases by age.

At 1–3 months after diagnosis, participants completed a structured questionnaire with content related to occupational history and other personal risk factors, including reproductive history, educational attainment, family history of breast cancer, age at menarche, smoking and alcohol consumption, body mass index, and home address (and duration of residence at that address) at time of diagnosis. Proxy respondents, who were close family members, completed a total of 75 of the questionnaires. Ethics committees at all participating hospitals and affiliated universities approved the protocol, and signed informed consent was obtained from participating subjects.

Occupational exposures were estimated using a standard methodology (Gérin et al. 1985; Siemiatycki 1990). Briefly, interviewers used a structured set of questionnaires and probed for details regarding each occupation that the subject had ever had, and a team of industrial hygienists and chemists attributed exposure to about 300 substances. For each substance, the team coded physical aspect, average duration of exposure in a working day, percentage of working days exposed during the period, confidence that there was actual exposure to each agent using a 4-point ordinal scale (probably no exposure, and “low,” “medium,” and “high” confidence of exposure), and level of intensity. Occupational exposures to four agents that may be associated with breast cancer, from the results of other analyses of our study (Labrèche et al. 2003, 2010), were included in the present analysis: organic solvents with reactive metabolites, extremely low-frequency magnetic fields, carbon monoxide, and PAHs from petroleum. Exposure indices were computed for exposures before 36 years of age (the period during which breast tissue may be more susceptible to exogenous insults), because female breast cells continue to develop until that age (as described in detail by Labrèche and Goldberg 1997).

Neighborhood deprivation may be a confounding factor in the association between breast cancer and air pollution because deprived populations often live in areas that are characterized by higher concentrations of air pollution (Crouse et al. 2009b; Jerrett et al. 2001). Thus, census data from 1996 were aggregated to the census tract level to describe socioeconomic characteristics of Montreal’s neighborhoods for assigning indicators of deprivation to subjects (Statistics Canada 2006). Variables describing median household income and percentage of adults who did not complete high school were compiled for the 350 census tracts that included addresses of subjects (heretofore referred to as neighborhood ecologic covariates).

### Assessment of exposure to traffic-related air pollution

A dense sampling of ambient NO_2_ was conducted in 2005 and 2006 (Crouse et al. 2009a, 2009b). NO_2_ is recognized as a marker of traffic-related pollution because of its collocational association with other pollutants (Beckerman et al. 2008). The locations for the samplers were selected using a location-allocation model that placed samplers in areas likely to have high spatial variability in traffic-related pollution and high population densities (Kanaroglou et al. 2005). Samplers were deployed at 133 locations across the Island of Montreal on three occasions (spring, summer, winter) for 2-week periods each time. The devices were Ogawa passive diffusion samplers (Ogawa and Co., Pompano Beach, FL, USA) that make use of triethanolamine-impregnated filters as an absorbent. Valid observations at 129 locations were obtained from all three sampling periods.

We used these observations to develop a land-use regression model to predict concentrations of mean annual NO_2_ for 2005–2006, at a resolution of 5 m across the island (Crouse et al. 2009a). We modeled the natural logarithm of NO_2_ on land-use and traffic-related variables to generate an exposure surface. The model explained 80% of the variability in concentrations of NO_2_. The residential addresses of cases and controls ascertained at the time of diagnosis (i.e., 1996) were linked to the exposure surface. Ninety-eight percent of subjects were geocoded to the *x*- and *y*-coordinates of their home address, and 2% were geocoded to the centroid of the area represented by the six-character postal code, which in Montreal refers usually to a block face or to a large apartment complex.

### Historical estimates of exposure

To account for the possibility that the spatial patterns have changed over time, we developed two separate but related methods (Chen et al. 2009, 2010) to extrapolate our exposure surface back to 1996 and to 1985 (i.e., approximately the time of diagnosis and 10 years before this, respectively) using measurements of NO_2_ from Environment Canada’s National Air Pollution Surveillance network [see Supplemental Material, Figure 1 (doi:10.1289/ehp.1002221)].

The network in Montreal included 13 fixed-site stations that were used to measure hourly concentrations of several criteria pollutants. Our goal was to use the spatial patterns of concentrations of NO_2_ collected at these stations to adjust our land-use regression surface to reflect the spatial patterns of the past. Because of incomplete information, however, observations of annual mean concentrations of NO_2_ were available at only nine stations for both 2006 and 1985 and at only 10 stations for both 2006 and 1996. We used inverse distance weighting on the mean annual concentrations at these stations, in each year, to interpolate spatial surfaces. Two approaches were used. First, we divided the interpolated surface describing concentrations of NO_2_ in 1985 by the interpolated surface for 2006. We then multiplied our original land-use regression model by this ratio to produce an extrapolated surface of estimated annual mean concentrations of NO_2_ for 1985. This process was repeated with the data for 1996. Second, we used the predicted values from our land-use regression surface in 2006 at the locations of the stations for which there were observations in 1985 (*n* = 10) and in 1996 (*n* = 12), respectively, and used these data to create new interpolated surfaces. Similar to the first method, we divided the new interpolated surface of the observed concentrations of NO_2_ in 1985 by the interpolated surface that made use of predicted values (from the land-use regression of 2006), which was then multiplied by the land-use regression surface of 2006. Again, the process was repeated with the data for 1996.

The key differences between these methods of adjustment is that the first is based on interpolated surfaces created with observed measurements of NO_2_ for 2006 but uses fewer data points, whereas the second is based on interpolated surfaces created with predicted estimates of NO_2_ for 2006 but is based on a greater number of data points.

We also created two additional surfaces that described the mean estimates of NO_2_ during the 10-year period between 1985 and 1996 (i.e., approximately the 10-year period before diagnosis). These final surfaces were created by adding each 1996 surface to the corresponding 1985 surface and dividing the outcome by 2. To assess the spatial variability between all seven exposure surfaces (i.e., land-use regression for 2006, two extrapolated surfaces for 1985, two extrapolated surfaces for 1996, two surfaces for 1985–1996), 1,000 randomly generated points were sampled and Pearson correlation coefficients were estimated.

### Statistical analysis

We used unconditional logistic regression to estimate ORs and associated 95% CIs. In order not to lose subjects because of missing values for continuous nonoccupational covariates, we used parametric smoothers (natural cubic splines) in age-adjusted logistic models to view the fitted response functions. Based on the fitted plots, we found cut points for each covariable that defined categories such that the OR within each category was approximately constant. Subjects with missing values were assigned to a “missing information” category. Only age at diagnosis and the two neighborhood ecologic covariates were treated as continuous variables.

Fully adjusted models included accepted and suspected risk factors for postmenopausal breast cancer (Table 1): age at diagnosis, family history, education, ethnicity, age at bilateral oophorectomy, age at menarche, age at first full-term pregnancy, alcohol consumption, and duration of hormonal replacement therapy. In addition, factors whose causal association with breast cancer is still uncertain were included as covariates: oral contraceptive use, smoking, total duration of breast-feeding, body mass index, neighborhood ecologic covariates, and the selected occupational exposures before 36 years of age. We also adjusted for the design variables proxy respondent status and for the hospital where subjects were diagnosed. Standard regression diagnostics were applied to identify possible influential subjects and to ensure that the models did not violate the assumptions of logistic regression.

We included NO_2_ as a continuous, linear variable after verifying this assumption through the use of natural cubic spline functions (2–3 degrees of freedom) and visual inspection of the fitted exposure–response curves. ORs are presented for each increase in exposure to NO_2_ of 5 ppb (referred to as OR_5ppb_) and for an increase across the interquartile range for each exposure period [presented in the Supplemental Material, Table 2 (doi:10.1289/ehp.1002221)]. The sensitivity of the results was assessed by analyses limited to those subjects for whom we had information on duration of residence and who had been residents at the same address for ≥ 10 years before diagnosis, by excluding proxy respondents, and by excluding controls with bladder cancer, because there is evidence that exposure to diesel exhaust may increase the risk of incidence of bladder cancer (Boffetta and Silverman 2001).

## Results

### Exposure surfaces

Concentrations of NO_2_ decreased over time, with the highest mean value observed in 1985 (20.1 ppb), and the lowest in 2006 (11.3 ppb) (Table 2). The trend of decreasing concentrations is consistent with observations from Environment Canada’s fixed-site stations [see Supplemental Material, Figure 2 (doi:10.1289/ehp.1002221)]. The seven surfaces reflected also a narrowing of the distributions of NO_2_ over time. We found positive correlations among 1,000 randomly sampled locations on the different exposure surfaces (*r* = 0.96–0.99) and among observed concentrations of annual mean concentrations of NO_2_ at the fixed-site stations for 1985, 1996, and 2005 (see Supplemental Material, Table 1), suggesting that the spatial patterns of NO_2_ did not vary importantly during the 20-year period between 1985 and 2006. Furthermore, the observed concentrations at the locations of the fixed-site monitoring stations in 2006 were correlated with those in 1996 (*r* = 0.89; 95% CI, 0.60–0.97) and in 1985 (*r* = 0.72; 95% CI, 0.12–0.94).

### Description of the cases and controls

**Figure 1:**
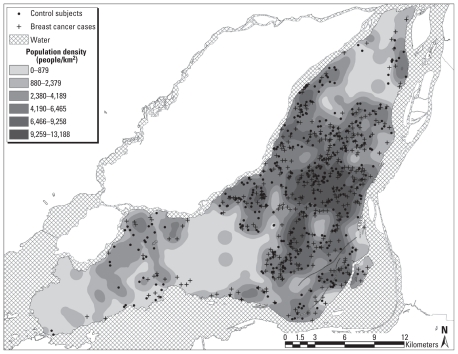
Spatial distribution of residential addresses of breast cancer cases and control subjects, Montreal, 1996–1997.

A total of 1,631 subjects were potentially eligible for this study. Interviews were conducted among 608 cases and 667 control subjects, for response rates of 81.1% for cases and 75.7% for controls. Of these 1,275 participants, 106 were deemed to be premenopausal and were therefore excluded; a further 79 participants had incomplete or inaccurate address information, and 291 subjects resided outside of the Island of Montreal. Therefore, these analyses included 799 subjects: 383 cases and 416 controls (Figure 1).

The most frequent sites of cancer in the 416 controls were colon (21.6%), uterus (19.0%), ovaries (9.1%), rectum (6.7%), and bladder (6.0%). We found associations for the generally accepted risk factors for postmenopausal breast cancer (i.e., family history, benign breast disease, education, age at menarche, duration of hormonal replacement therapy) (Table 2). We found essentially no differences in characteristics between the home neighborhoods of the cases and controls (i.e., median household income and percentage of adults ≥ 15 years of age without high school diploma were C$34,239 and C$34,436, and 32.6% and 32.8%, respectively, for cases and controls).

### Associations between postmenopausal breast cancer and air pollution

In the age-adjusted models, the OR_5ppb_ ranged from a low of 1.05 (95% CI, 0.91–1.22) using estimates of exposure for 1985 to a high of 1.15 (95% CI, 0.89–1.48) in 2006 (Table 3). In the fully adjusted model using estimates of exposure from 1996, the OR_5ppb_ was 1.31 (95% CI, 1.00–1.71). The ORs calculated per interquartile range were less variable between exposure periods compared with those computed per 5 ppb, varying from 1.19 in 1985 to 1.30 in 1996 [see Supplemental Material, Table 2 (doi:10.1289/ehp.1002221)]. The two methods of historical extrapolation produced almost identical associations with the risk of developing invasive breast cancer (Table 3; see also Supplemental Material, Table 2).

### Sensitivity analyses

Adjustment for individual-level educational status had the strongest effect on the size of the ORs. For example, in a reduced model using estimates of exposure for 2006, adjusted only for age and individual-level educational status, the OR_5ppb_ was 1.21 (95% CI, 0.94–1.57). None of the other covariates included in the fully adjusted models contributed > 3% to the increase in the ORs.

We also computed ORs for the 408 women (195 cases, 213 controls) who reported that they had been residents at the same address for at least 10 years before diagnosis. These ORs were slightly larger than those produced with the full study population, although because of the smaller sample size, the CIs were wider (Table 3). For example, in the fully adjusted models, the OR_5ppb_ ranged from a low of 1.23 (95% CI, 0.87–1.74) using estimates for 1985 to a high of 1.52 (95% CI, 0.82–2.81) using estimates for 2006. Similar to the findings with the full data set, the ORs calculated per interquartile range were much less variable between exposure periods, varying from 1.31 (95% CI, 0.82–2.09) to 1.35 (95% CI, 0.84–2.18) [see Supplemental Material, Table 2 (doi:10.1289/ehp.1002221)].

We also computed ORs for subsets that excluded proxy respondents, whose interviews could have elicited less accurate information (362 cases, 362 controls), and controls diagnosed with bladder cancer, a cancer linked to diesel exhaust fumes exposure (383 cases, 392 controls). In both analyses, we found associations very similar to those found with the full study population [see Supplemental Material, Table 3 (doi:10.1289/ehp.1002221)].

## Discussion

We found evidence of an association between exposure to outdoor concentrations of NO_2_ and the incidence of postmenopausal breast cancer. Although the size of effect varied somewhat using estimates of exposure from different periods, we found an increased risk of approximately 25% for every increase of 5 ppb in exposure.

Several methodological strengths of our study suggest that the effect sizes are unbiased and perhaps even conservative. Response rates among cases and controls were relatively high, and there is no reason to believe that catchment areas of different hospitals would differ for cases and controls by level of exposure to traffic-related pollution, thus limiting the likelihood of selection bias. The selection of cancer patients as controls reduces the possibility of recall bias for the covariates, and we estimated concentrations of NO_2_ using independent data sources. The cancer sites in the control group are not known or suspected to be associated with air pollution, with the possible exception of bladder cancer. However, should any of the control series cancer sites be shown subsequently to be causally associated with air pollution, this would have the effect of attenuating the risks demonstrated in our study. The exclusion of bladder cancer, which has been associated with diesel exhaust fumes, did not substantially change the results. Additionally, we found that individual-level educational attainment was the only covariate to affect the estimate of risk by ≥ 5%.

We acknowledge that the present analysis provides only partial information on personal exposure to air pollution. Total personal exposure relates to a number of factors, including daily activity patterns and amount of time spent indoors and outdoors, among others. Two limitations of using the home address as a surrogate of exposure are related to population mobility: People do not necessarily live in the same home over the course of their lifetime, nor do they spend all of their time at home. Although it is true that many subjects may spend their days away from home, a study by Leech et al. (2002) found that Canadian adults spend on average approximately 67–68% of their time at home (indoors and outdoors combined). The finding of higher risks among subjects who lived for ≥ 10 years at the same address before diagnosis suggests that our risk estimates may be conservative.

Last, given the inherent imprecision associated with geocoded addresses and other geographic data, as well as the fact that we used spatially derived exposures as surrogates for personal exposures, the risk estimates presented here are likely subject to nondifferential misclassification bias. Our results thus probably underestimate the true estimates of the relative risk of postmenopausal breast cancer associated with exposure to air pollution in this population.

Our findings should not be interpreted as meaning that NO_2_ is a causal factor; it is more likely a marker of the complex mixture that is derived from combustion (Goldberg 2007).

We found an association between exposures to traffic-related air pollution and the incidence of postmenopausal breast cancer in a city that by international standards is relatively unpolluted. Our findings are qualitatively similar to those reported in the two other studies that examined the hypothesis that breast cancer may be associated with exposure to air pollution (Bonner et al. 2005; Nie et al. 2007). Our results differ somewhat, however, from those studies. Among postmenopausal women, those authors found associations between early-life exposures to markers of air pollution and the incidence of breast cancer, but no associations with exposures 10 and 20 years before diagnosis. We could not assess associations with early-life exposures, nor do we know what age periods may be critical in the induction of cancer. Our analysis of occupational exposures (Labrèche et al. 2010) suggests that exposures to some compounds before 36 years of age may be more important. If this is the case, it is possible that the risks observed here are underestimated. Studies are needed to verify whether these results represent true associations or whether they are attributable to chance or to undetected bias. If these associations are verified, additional studies should explore potential critical periods of exposure to air pollution in relation to the development of breast cancer.

## Figures and Tables

**Table 1:** Distribution of risk factors and age-adjusted ORs and 95% CIs, postmenopausal breast cancer, Montreal, Canada (*n* = 799).

Variable (reference category)^a^	Cases [*n* (%)]	Controls [*n* (%)]	OR (95% CI)
Mother or sister with breast cancer (no)	210 (54.8)	279 (67.1)	1
Yes	83 (21.7)	47 (11.3)	2.36 (1.58–3.52)
No for mother, no sisters	79 (20.6)	76 (18.3)	1.40 (0.97–2.01)
Missing information	11 (2.9)	14 (3.4)	1.13 (0.50–2.55)
Oophorectomy (never)	276 (72.1)	194 (46.6)	1
Only one ovary removed	27 (7)	30 (7.2)	0.61 (0.35–1.07)
Age at bilateral oophorectomy, years			
< 44	26 (6.8)	34 (8.2)	0.55 (0.32–0.95)
45–49	19 (5)	15 (3.6)	0.89 (0.44–1.79)
50–54	16 (4.2)	26 (6.3)	0.39 (0.20–0.76)
≥ 55	17 (4.4)	106 (25.5)	0.12 (0.07–0.20)
Missing information	2 (0.5)	11 (2.6)	0.13 (0.03–0.59)
Education, years (≤ 7)	107 (27.9)	154 (37)	1
8–10	78 (20.4)	107 (25.7)	1.05 (0.72–1.54)
11–17	178 (46.5)	138 (33.2)	1.82 (1.30–2.53)
≥ 18	20 (5.2)	17 (4.1)	1.58 (0.78–3.17)
Ethnicity (French)	232 (60.6)	219 (52.6)	1
English and others	98 (25.6)	110 (26.4)	0.86 (0.62–1.20)
Jewish and Italian	53 (13.8)	87 (20.9)	0.58 (0.39–0.86)
Age at menarche, years (≥ 16)	24 (6.3)	39 (9.4)	1
14–15	97 (25.3)	109 (26.2)	1.47 (0.82–2.62)
13	110 (28.7)	100 (24)	1.82 (1.02–3.24)
12	78 (20.4)	98 (23.6)	1.28 (0.71–2.30)
≤ 11	74 (19.3)	70 (16.8)	1.65 (0.90–3.02)
Age at first full-term pregnancy, years (never)	83 (21.7)	77 (18.5)	1
< 18	15 (3.9)	12 (2.9)	1.12 (0.49–2.55)
≥ 18 to 26	166 (43.3)	214 (51.4)	0.71 (0.49–1.04)
> 26 to 30	59 (15.4)	56 (13.5)	1.00 (0.62–1.62)
> 30	46 (12)	36 (8.7)	1.24 (0.72–2.12)
Pregnant but never full term	14 (3.7)	21 (5)	0.59 (0.28–1.24)
Breast-feeding, duration, weeks (0)	296 (77.3)	303 (72.8)	1
0–80	69 (18)	77 (18.5)	0.93 (0.65–1.34)
> 80	18 (4.7)	36 (8.7)	0.53 (0.30–0.96)
Years of oral contraception use (never)	248 (64.8)	290 (69.7)	1
< 1	42 (11)	25 (6)	1.78 (1.04–3.05)
≥ 1	93 (24.3)	101 (24.3)	0.96 (0.67–1.38)
Hormone replacement therapy, months (0)	170 (44.4)	236 (56.7)	1
1–19	53 (13.8)	57 (13.7)	1.28 (0.84–1.95)
20–44	29 (7.6)	34 (8.2)	1.14 (0.67–1.96)
45–74	35 (9.1)	21 (5)	2.19 (1.23–3.92)
75–99	16 (4.2)	13 (3.1)	1.58 (0.73–3.39)
≥ 100	80 (20.9)	55 (13.2)	1.98 (1.33–2.95)
Body mass index (18.5 to < 25)	190 (49.6)	194 (46.6)	1
25 to < 30	123 (32.1)	133 (32.0)	0.97 (0.71–1.34)
> 30 to < 35	48 (12.5)	51 (12.3)	1.00 (0.64–1.56)
≥ 35	15 (3.9)	28 (6.7)	0.54 (0.28–1.06)
< 18.5	6 (1.6)	9 (2.2)	0.69 (0.24–1.99)
Missing information	1 (0.3)	1 (0.2)	NE
Tobacco exposure (none)^b^	45 (11.7)	52 (12.5)	1
Environmental tobacco smoke only	160 (41.8)	148 (35.6)	1.17 (0.66–2.08)
Active smoker with or without exposure to environmental tobacco smoke	176 (46)	209 (50.2)	1.02 (0.65–1.61)
Missing information	2 (0.5)	7 (1.7)	NE
Respondent (self)	362 (94.5)	362 (87)	1
Proxy	21 (5.5)	54 (13)	0.41 (0.24–0.69)
Alcohol status (never drinker)^c^	193 (50.4)	229 (55)	1
Former drinker	50 (13.1)	55 (13.2)	1.01 (0.66–1.57)
Infrequent drinker	53 (13.8)	53 (12.7)	1.15 (0.75–1.76)
Current drinker	87 (22.7)	79 (19)	1.26 (0.88–1.81)
Benign breast disease (no)	197 (51.4)	333 (80)	1
Yes	185 (48.3)	83 (20)	3.71 (2.70–5.08)
Missing information	1 (0.3)	0 (0)	NE
Occupational exposure to solvents with reactive metabolites (not exposed)^d^	338 (88.3)	374 (89.9)	1
Nonsubstantial, 5 years	5 (1.3)	4 (1)	1.35 (0.36–5.09)
Substantial, 5 years	6 (1.6)	5 (1.2)	1.26 (0.38–4.17)
Exposed only at *R* = 1	12 (3.1)	9 (2.2)	1.43 (0.60–3.45)
Other exposures	22 (5.7)	24 (5.8)	0.99 (0.55–1.81)
Occupational exposure to extremely low magnetic fields (not exposed)^d^	81 (21.1)	102 (24.5)	1
Nonsubstantial, 5 years	107 (27.9)	96 (23.1)	1.30 (0.86–1.97)
Substantial, 5 years	48 (12.5)	44 (10.6)	1.30 (0.78–2.17)
Exposed only at *R* = 1	8 (2.1)	18 (4.3)	0.57 (0.24–1.37)
Other exposures	139 (36.3)	156 (37.5)	1.06 (0.73–1.55)
Occupational exposure to carbon monoxide (not exposed)^d^	299 (78.1)	339 (81.5)	1
Nonsubstantial, 5 years	36 (9.4)	21 (5.0)	1.87 (1.07–3.27)
Substantial, 5 years	0 (0.0)	0 (0.0)	NE
Exposed only at *R* = 1	1 (0.3)	1 (0.2)	1.18 (0.07–19.10)
Other exposures	47 (12.3)	55 (13.2)	0.94 (0.62–1.43)
Occupational exposure to polycyclic aromatic hydrocarbons from petroleum (not exposed)^d^	354 (92.4)	386 (92.8)	1
Nonsubstantial, 5 years	9 (2.3)	4 (1.0)	2.34 (0.72–7.63)
Substantial, 5 years	2 (0.5)	2 (0.5)	1.07 (0.15–7.69)
Exposed only at *R* = 1	2 (0.5)	8 (1.9)	0.28 (0.64–1.22)
Other exposures	16 (4.2)	16 (3.8)	1.03 (0.51–2.10)

NE, not estimated.

a Categories shown are those modeled in the analyses (see Table 3); reference group for each category is in parentheses.

b This variable was compiled with information acquired from several questions. Subjects were asked if they had smoked 100 cigarettes over the course of their lifetime, along with follow-up questions, to determine whether they were former, current, or never-smokers. Subjects were also asked whether they had ever been exposed to residential or occupational environmental tobacco smoke, along with follow-up questions concerning duration and kind of exposure.

c The categories for this variable were determined based on information from several questions. Subjects were asked whether there had ever been a time in their life when they had consumed one or more drinks of beer, wine, or liquor (respectively) on a monthly basis, or on a weekly basis.

d Substantial exposure, ≥ 5 years of exposure at medium or high levels of intensity; nonsubstantial exposure, < 5 years of exposure at medium or high levels of intensity, but still ≥ 5 years of exposure at any intensity; exposed only at *R* = 1, exposure only at the lowest level of confidence; other exposures, exposures totaling < 5 years.

**Table 2:** Distributions of concentrations of NO_2_ (ppb) in seven different exposure surfaces, Island of Montreal, Canada.

Model year	Minimum	25th percentile	Mean	75th percentile	Maximum	Median
2006	4.3	9.2	11.3	12.9	37.4	10.8
1996^a^	6.0	12.9	15.6	17.8	44.5	15.1
Mean of 1985 and 1996^a^	6.9	14.8	17.9	20.3	55.6	17.2
1985^a^	7.8	16.5	20.1	22.8	66.8	19.3
1996^b^	4.9	10.3	12.7	14.7	39.5	12.2
Mean of 1985 and 1996^b^	5.6	11.6	14.3	16.6	49.3	13.8
1985^b^	6.2	12.7	15.8	18.3	59.2	15.1

a Extrapolated using observed concentrations of NO_2_ at each fixed-site monitoring station.

b Extrapolated using predicted concentrations of NO_2_ derived from the land-use regression in 2006 at each fixed-site monitoring station.

**Table 3:** Associations between ambient concentrations of NO_2_ (per 5 ppb) and postmenopausal breast cancer, Island of Montreal, Canada.

	Full data set (*n* = 799; 383 cases, 416 controls)		Limited to subjects who were residents at the same address for at least 10 years before interview (*n* = 408; 195 cases, 213 controls)	
	Age adjusted	Fully adjusted^a^	Age adjusted	Fully adjusted^a^
Exposure surface	OR (95% CI)	OR (95% CI)	OR (95% CI)	OR (95% CI)
2006	1.15 (0.89–1.48)	1.35 (0.94–1.94)	1.08 (0.75–1.56)	1.52 (0.82–2.81)
1996^b^	1.14 (0.91–1.42)	1.36 (0.99–1.88)	1.05 (0.76–1.45)	1.42 (0.81–2.48)
Mean of 1996 and 1985^b^	1.10 (0.90–1.34)	1.25 (0.94–1.65)	1.06 (0.80–1.41)	1.34 (0.83–2.16)
1985^b^	1.07 (0.90–1.27)	1.17 (0.91–1.50)	1.06 (0.83–1.36)	1.28 (0.84–1.93)
1996^c^	1.10 (0.91–1.32)	1.31 (1.00–1.71)	1.02 (0.78–1.34)	1.34 (0.84–2.14)
Mean of 1996 and 1985^c^	1.07 (0.91–1.27)	1.22 (0.97–1.54)	1.04 (0.82–1.32)	1.28 (0.86–1.91)
1985^c^	1.05 (0.91–1.22)	1.16 (0.94–1.42)	1.04 (0.85–1.29)	1.23 (0.87–1.74)

a Adjusted for hospital of diagnosis, mother or sister with breast cancer, oophorectomy, years of education, ethnicity, age at menarche, age at first full-term pregnancy, breast-feeding history, oral contraceptive use, hormone replacement therapy use, body mass index, exposure to tobacco smoke, respondent/proxy status, alcohol consumption, history of benign breast disease, and occupational exposures to solvents with reactive metabolites, extremely low magnetic fields, carbon monoxide, and PAHs; and two neighborhood ecologic covariates: median household income and percentage of adults without a high school diploma. See Table 1.

b Extrapolated using observed concentrations of NO_2_ at each fixed-site monitoring station.

c Extrapolated using predicted concentrations of NO_2_ derived from the land-use regression in 2006 at each fixed-site monitoring station.
